# The relationship between family and residential settings: Aging in place vs long term care facility in South Korea

**DOI:** 10.1093/geroni/igab046.2366

**Published:** 2021-12-17

**Authors:** Joohong Min, Seung-eun Oh, Bon Kim

**Affiliations:** 1 Jeju National University, Jeju, Cheju-do, Republic of Korea; 2 Jeju National University, Jeju National University, Cheju-do, Republic of Korea; 3 Jeju National University, Jeju-si, Cheju-do, Republic of Korea

## Abstract

Objective: This study aims to explore the factors influencing long-term-care beneficiaries’ decisions between home health care and institutional care in South Korea. This study further investigated the association between these two types of long-term care services and emotional, financial, and physical burden alleviation among the beneficiaries and their family members. Methods: We analyzed data from 2019 Long-Term Care Survey. Out of 5,606 respondents, 4,079 long-term care beneficiaries and family members were included in this study. Logistic regression models were conducted to understand factors associated with types of long-term care services, and the association between types of long-term care services and burden alleviation. Results: Having a spouse, having children, or a shorter geographic distance between the beneficiaries and their family members were factors associated with higher likelihood of receiving home health care rather than institutional care. Also, more sever mobility limitation, outpatient care, and fall experiences were associated with higher likelihood of receiving institutional care. Utilization of institutional care was significantly associated with higher alleviation of physical burden. No significant differences between home health care and institutional care were found on emotional and financial burden alleviation. Conclusions: The findings suggest that family resources may be beneficial for older adults to pursue aging in place in later life. However, the physical toll of their family members may exist. Our findings provide evidence to inform public policy decisions on long-term care services for older adults and their family members.

